# Laparoscopic surgery for two patients with strangulated transomental hernias

**DOI:** 10.1186/s40792-020-00815-y

**Published:** 2020-03-18

**Authors:** Yuka Fujimoto, Yuki Ohya, Shintaro Hayashida, Masayoshi Iizaka, Yuto Maeda, Sayahito Kumamoto, Akira Tsuji, Hidekatsu Shibata, Kunitaka Kuramoto, Hironori Hayashi, Osamu Nakahara, Shinjiro Tomiyasu, Yukihiro Inomata

**Affiliations:** Department of Surgery, Kumamoto Rosai Hospital, 1670 Takehara-machi, Yatsushiro, Kumamoto, 866-8533 Japan

**Keywords:** Transomental hernia, Internal hernia, Laparoscopic surgery, Intestinal viability, Omentum

## Abstract

**Background:**

Transomental hernias are a rare type of internal hernia. We report two cases of successful cases of laparoscopic repair. One required laparotomy due to concern for intestinal viability.

**Case presentation:**

The first patient was a 67-year-old man who presented with abdominal pain and vomiting. He had no history of laparotomy or abdominal injury. Computed tomography suggested small bowel obstruction and possible intestinal strangulation. Emergent laparoscopy found approximately 200 cm of small bowel was strangulated around the greater omentum. The strangulation was released laparoscopically, but because of the color of the strangulated bowel, laparotomy was performed to evaluate viability. The involved portion of intestine was not resected. The patient experienced transient postoperative paralytic ileus and was discharged on postoperative day 14. The second patient was a 56-year-old man who presented with abdominal pain. Abdominal computed tomography revealed dilatation of the small intestine and a closed loop suggesting ileus due to intestinal strangulation. An emergency laparoscopy found a transomental hernia, and the strangulation was released laparoscopically. Recovery was uneventful, and the patient was discharged on postoperative day 6.

**Conclusion:**

Transomental hernia can be successfully treated laparoscopically. In cases where bowel viability is a concern, laparotomy should not be hesitated.

## Background

Transomental hernia is a rare cause of small bowel obstruction that may occur in patients without a history of abdominal surgery or trauma. It is an internal hernia in which the intestine is inserted into a transomental hiatus that is congenital or the result of trauma or atrophy of connective tissue. The incidence of internal hernias has been estimated as 0.2–0.9% in autopsy studies. Transomental hernias involving the greater or lesser omentum are rare, accounting for approximately 1–4% of internal hernias [1–3]. Preoperative diagnosis is difficult because of a lack of specific clinical findings. Prompt laparoscopic surgery is effective for treating internal hernias and spontaneous transomental hernias without evidence of necrosis or perforation of the hernial contents [3, 4]. We report consecutive two cases of transomental hernia through the greater omentum without any history of abdominal surgery or trauma. The incarcerated small bowel was released laparoscopically in both patients but the first case was switched to a laparotomy to evaluate the viability of the strangulated intestine.

## Case presentation

### Case 1

**Fig. 1:**
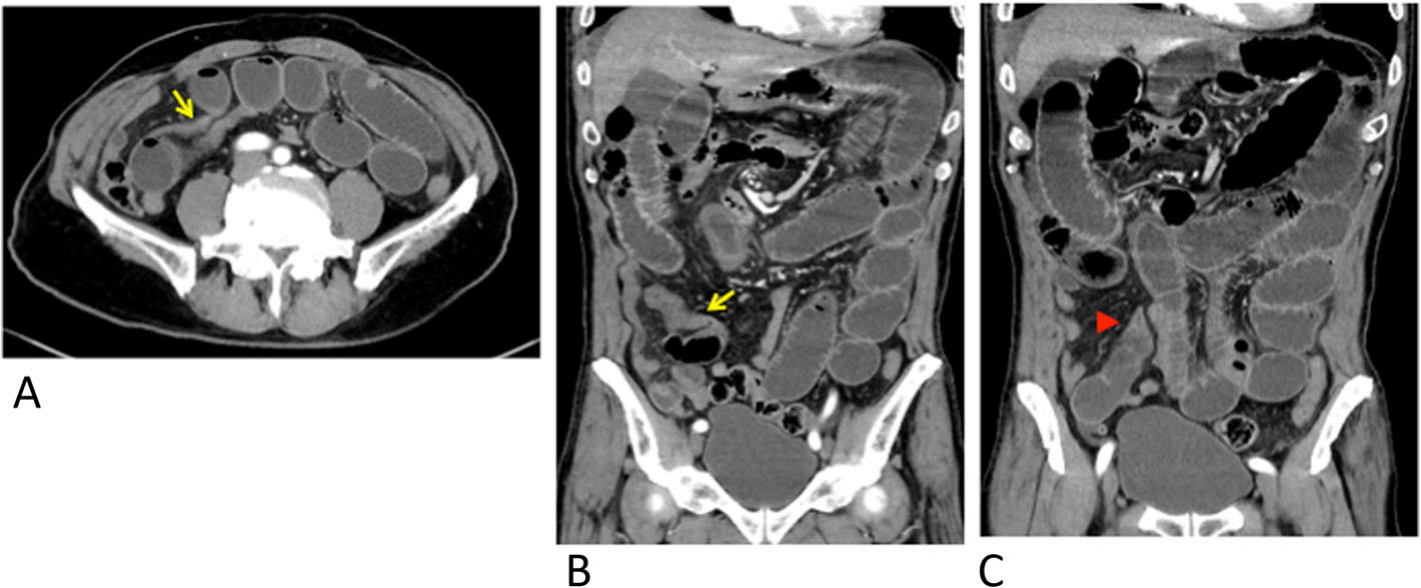
Enhanced abdominal computed tomography with arrows showing the border of the dilated segment of intestine (**a**, **b**) and an arrowhead indicating a fat notch (**c**)

**Fig. 2:**
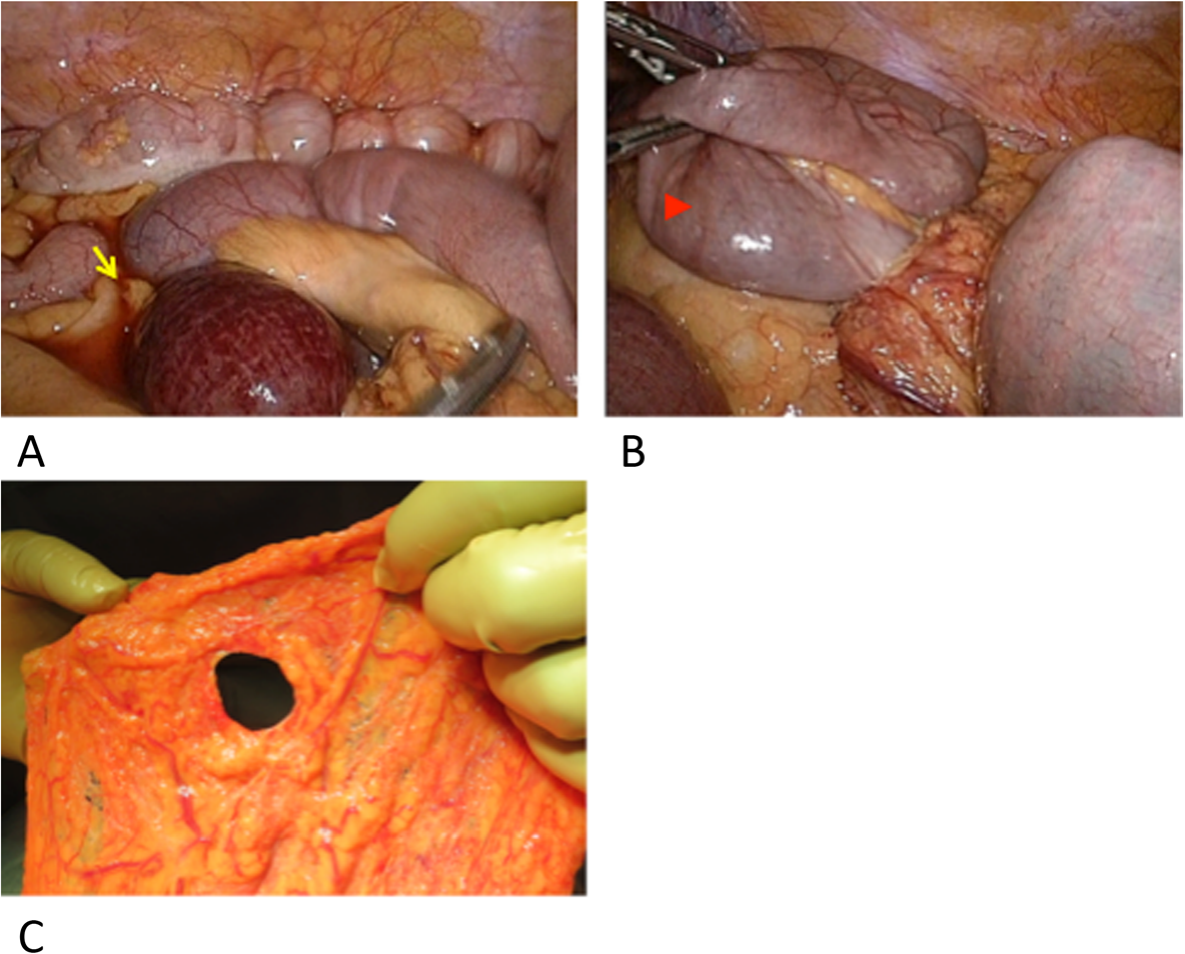
Intraoperative images showing hemorrhagic ascites and the strangulated portion (arrow) of the small bowel (**a**), the incarcerated small bowel (arrowhead) (**b**), and the omentum with a 3-cm diameter hole (**c**)

A 67-year-old man with a history of hypertension and diabetes presented with abdominal pain, bloating, and vomiting. He had no history of abdominal injury or abdominal surgery. There were no signs of peritoneal irritation, and he had no fever. Blood tests showed mild dehydration, and the initial laboratory evaluation revealed no abnormalities, but indicated a minimal inflammatory reaction with a white blood cell count of 10.4 K cells/mm3 (normal 3.9–9.8) and C-reactive protein of 0.06 mg/dL (normal 0.0–0.3). Plain abdominal computed tomography (CT) revealed dilatation and content retention in the small intestine, but the origin of the obstruction was not clear. The diagnosis was small bowel obstruction, and the patient was admitted for fasting. The abdominal tenderness increased, and abdominal contrast-enhanced computed tomography (CE-CT) on the day after hospitalization showed small bowel obstruction with a fat notch sign that suggested strangulation ileus caused by an internal hernia (Fig. 1). Emergency laparoscopic surgery was conducted with three ports, a 10-mm umbilical port, a 10-mm left lower quadrant port, and a 5-mm lower midline port. Laparoscopy revealed a small amount of hemorrhagic ascites and that the small intestine had herniated through an abnormal hiatus of the greater omentum and was strangulated. Approximately 200 cm of strangulated small bowel was laparoscopically reduced and replaced in the abdominal cavity without injury. It was difficult to judge the viability of the intestine by the color only under the laparoscope (Fig. 2), and the procedure was converted to the laparotomic approach. A laparotomy, with an 8-cm midline incision, allowed visualizing and palpating the entire length of the involved intestine and the mesentery. Peristalsis was seen, and there was no evidence of necrosis of any part of the intestinal wall. The intestinal tract was preserved unresected. A defect with 3 cm in diameter of the omentum was closed with continuous suture through the laparotomy wound. The total operative time was 168 min. Postoperative paralytic ileus resolved in 5 days of conservative treatment. Oral intake resumed on postoperative day 13, and a postoperative abdominal CE-CT confirmed intact blood supply to the intestine. The patient was discharged on postoperative day 14.

### Case 2

**Fig. 3:**
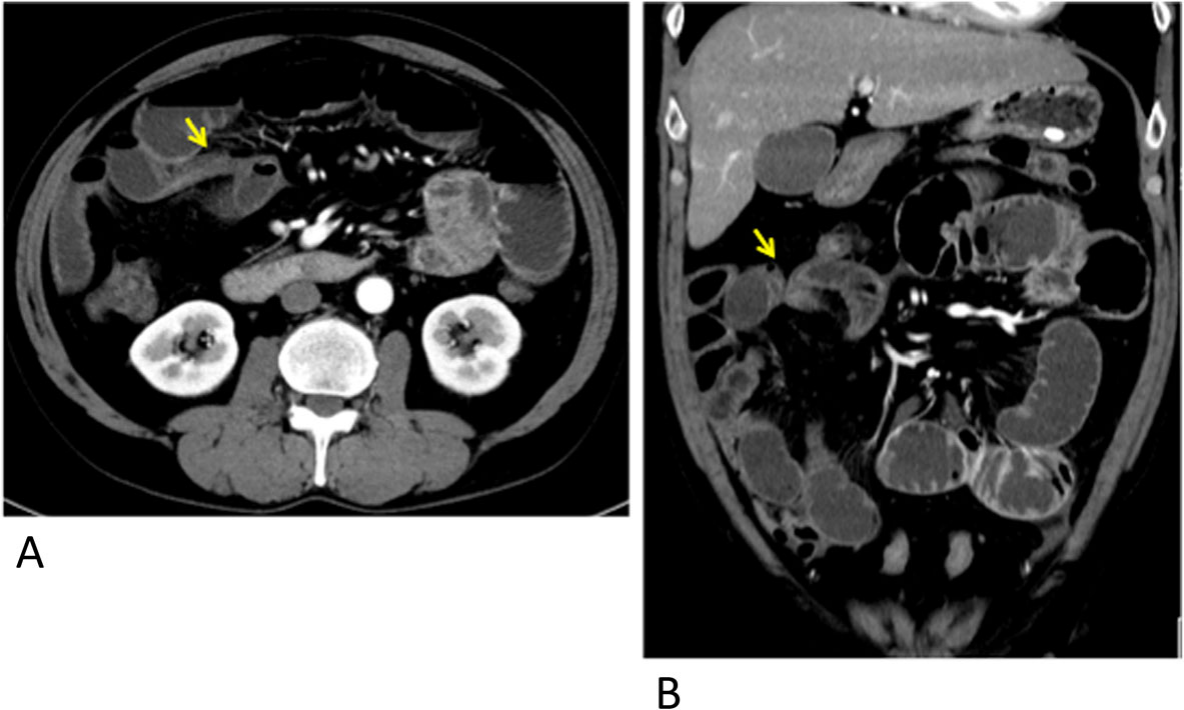
Enhanced abdominal computed tomography showing two caliber changes (arrows) that indicate a closed loop (**a**, **b**)

**Fig. 4:**
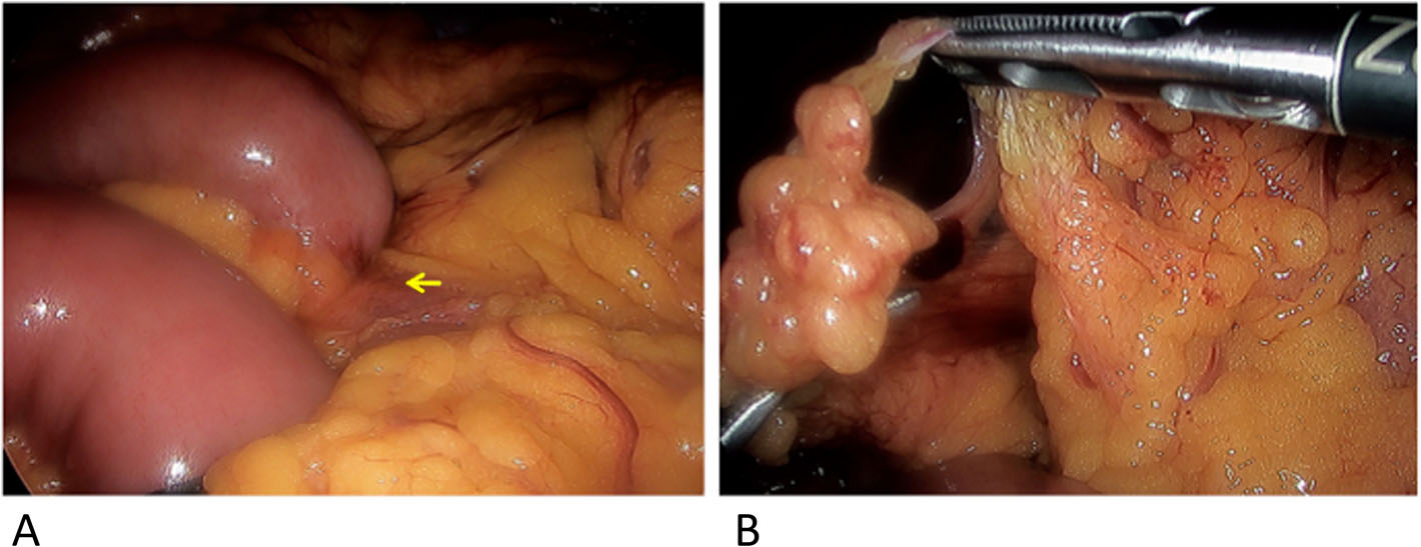
Intraoperative images showing the incarcerated small bowel in the upper right abdomen (arrow) (**a**). The omentum had a defect with 4 cm in diameter (**b**)

A 56-year-old man presented with acute abdominal pain and bloating after 12 months from the first case. He had no history of laparotomy or abdominal injury. There were no signs of peritoneal irritation and no fever. Blood tests showed a mild inflammatory response with increase of white blood cell count of 14.4 K cells/mm^3^ (normal 3.9–9.8) and C-reactive protein of 0.51 mg/dL (normal 0.0–0.3). Abdominal CE-CT revealed dilatation of the small intestine and a closed loop suggesting strangulated ileus (Fig. 3). Emergency laparoscopic surgery revealed that approximately 10 cm of small intestine was incarcerated through an omental defect of 4 cm in diameter (Fig. 4). The involved portion of the small intestine could be judged not necrotic under the laparoscopy with confidence. As the defect was at the edge of the omentum, the surrounding rim was transected to eliminate the hole laparoscopically. The total operative time was 31 min. Recovery was uneventful and the patient was discharged on postoperative day 6.

## Discussion

A transomental hernia is a rare cause of Not only in virgin cases small bowel obstruction that is occasionally diagnosed during surgery [1–6]. Preoperative diagnosis of transomental hernia is difficult, but abdominal CT can help to detect strangulation ileus resulting from transomental hernia as in these two cases [1–4]. A transomental hernia shows finding of small bowel obstruction. In our two cases, transverse colon loops were located posterior to dilated intestinal loops, as previously reported [7]. Identification of intestinal localization was easier by using the CE-CT. That was why we used the CE-CT as the initial imaging study for case 2. This finding might be specific and useful for the diagnosis of transomental hernia. Because the necessity for emergency surgery was not clearly informed in the first plain CT in case 1, the patient was initially treated conservatively. Emergency surgery is usually recommended for cases of small bowel obstruction in patients with a virgin abdomen [8]. Earlier laparoscopic surgery following the earlier diagnosis might have avoided the conversion to laparotomy in the first case.

The best approach for the surgery of small bowel obstruction with strangulation is controversial. In case 1, the color of the intestinal tract under the observation by laparoscopy led to a concern of its viability. That was why the procedure was switched to a laparotomy. The viability of a strangulated intestine can be judged by the color and the presence of swelling resulting from ischemia and compression of the intestinal wall. Palpation with gazing for pulsation of the small mesenteric arteries and tactile evaluation of the wall thickness are possible only under the laparotomy. Visible light spectrophotometry and laser Doppler flowmetry can help to determine whether bowel preservation is possible without a need of laparotomy [6]. The use of indocyanine green to evaluate intestinal blood flow during surgery for incarcerated hernia has also been reported [9]. If such method for evaluating intestinal blood flow had been available, the laparotomy might be unnecessary in case 1. In case 2, laparotomy was not needed to confirm the viability of the involved portion of intestine, because the involved length was shorter and the discoloration of the intestine was milder than the case 1.

The laparoscopic approach to small bowel obstruction has been expanded these days [10]. Based on our experience, laparoscopic surgery should be applied even in cases with strangulation unless there are clear preoperative signs of perforation or necrosis. We can expect the completion of the procedure without conversion to laparotomy like in case 2. Usage of sophisticated instruments for detection of blood supply to the intestine might increase the possibility of complete laparoscopic surgery in those cases. Not only in virgin cases but also in cases with positive history of surgery or trauma, laparoscopic approach can be applied unless the patient has definite contraindications [11]. Of course, in that situation, we should not hesitate to convert to laparotomy if there is a concern about the complications.

## Conclusions

We report two cases of strangulated transomental hernia to which laparoscopic surgery was applied. Although strangulated small bowel was released laparoscopically in both cases, it was difficult to confirm the viability of the intestine by laparoscopy in the first case. Our experience showed laparoscopic approach should be applied even in strangulated hernia. However, in cases that necrosis cannot be ruled out under the laparoscopy, conversion to laparotomy should not be hesitated.

## Data Availability

Not applicable

## Abbreviations

CT: Computed tomography
CE-CT: Contrast-enhanced computed tomography
